# Thyroglossal duct cyst carcinoma with concurrent thyroid carcinoma: a case report

**DOI:** 10.1186/1752-1947-2-132

**Published:** 2008-04-29

**Authors:** Vittorio Gebbia, Carlo Di Gregorio, Marco Attard

**Affiliations:** 1 Department of Experimental Oncology and Clinical Applications, University of Palermo, Italy; 2 Medical Oncology Unit, La Maddalena Clinic for Cancer, Palermo, Italy; 3 Service of Plastic Surgery, Casa di Cura Candela, Palermo, Italy; 4 Endocrinology Operative Unit, Ospedale 'V Cervello', Palermo, Italy

## Abstract

**Introduction:**

Thyroglossal duct carcinoma is a very rare finding and its presentation is similar to that of a benign cyst, which is a relatively common developmental abnormality that may manifest as a midline, neck mass. In general the diagnosis of thyroglossal duct carcinoma is based on the pathologic examination of the mass, but needle aspiration cytology, ultrasound and computed tomography play a role in the differential diagnosis of malignancy.

**Case presentation:**

A further case of thyroglossal duct carcinoma and concurrent thyroid carcinoma with locoregional lymph node metastases affecting a 40-year-old woman followed up for 4 years is presented and discussed.

**Conclusion:**

Sistrunk's surgical technique must always be the initial treatment, but in case of carcinoma further surgery, that is, thyroidectomy with or without lymph node dissection, and treatment with radioactive iodine have to be considered according to the microscopic and clinical findings. Accurate pre-operative clinical and radiological evaluation should be performed in order to plan surgical strategy.

## Introduction

A thyroglossal duct cyst is a developmental abnormality of the thyroid gland, which often manifests clinically as a mass along the thyroglossal tract [1]. The thyroglossal tract is a remnant of the thyroid development. During its descent, in fact, the thyroid gland connects to the base of the tongue through this duct, which later involutes and disappears. Sometimes it may persist and undergo dilatation responsible for thyroglossal duct cyst formation. The presence of ectopic thyroid tissue in a thyroglossal duct cyst varies from 1.5% to 45% of cases [1] and can be explained on the basis of the gland embryology [2].

Carcinoma arising in this tract is an uncommon clinico-pathologic entity [3,4]. The median age at presentation is 40 years [3,4] and females are affected slightly more often than males with a 3:2 ratio. The peak incidence for women and men is in the third and sixth decades, respectively. Overall age and sex distribution is similar to that of patients with thyroid carcinoma. Regional lymph node metastases of papillary carcinomas in thyroglossal cysts occur much less frequently than in primary cancer of the thyroid, with incidence of less than 8% of cases [1]. Moreover, papillary thyroglossal duct carcinoma (TDC) seldom shows distant metastases and its prognosis is similar to that of the thyroid papillary carcinomas.

Although a few cases of follicular, mixed follicular-papillary, squamous cell and other types of carcinomas have been reported [5,6], most tumors are histologically papillary carcinomas and medullary variants have not been reported [7]. Association with papillary carcinoma in the thyroid gland has been observed in about 25–40% of patients in whom the gland was removed as a part of surgical treatment [4-6].

Presentation is similar to that of a benign thyroglossal duct cyst. A rapid increase in growth or the presence of a firm palpable mass may be signs of malignancy, but diagnosis is often based on the pathologic analysis of the resection in most of the reports [8]. Fine-needle aspiration may confirm the presence of TDC in only a fraction of cases [9,10], while computed tomography (CT) scan, neck nuclear magnetic resonance (NMR) spectroscopy and ultrasounds may show a solid mass with invasive features and therefore play an important staging role in pre-operative diagnosis of malignancy, its extension and then in the planning of primary treatment [11-13].

## Case presentation

**Figure 1:**
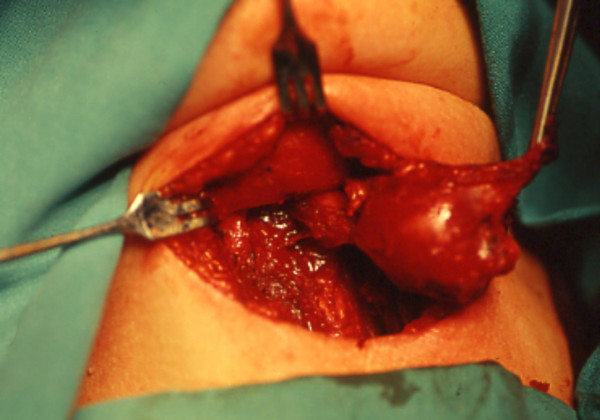
**Sistrunk's surgical procedure**. A mass of 5 cm was removed including the entire duct from the gland to the level of the foramen cecum and the middle portion of the hyoid bone.

A 40-year-old previously healthy woman was admitted to the Service of Plastic Surgery in February 2000 because of an enlarging anterior midline neck mass she had noted some months before. In the previous 2 months the neck mass had been rapidly growing in size. Medical history included a cerebral trauma owing to a car accident, depression treated with fluoxetine and uterine fibroma. The patient had not been previously exposed to radiation or other known carcinogens. Familiar medical history was negative for thyroid gland or neoplastic diseases. Physical examination revealed a painless well-demarcated mass of about 6 cm localized in the midline of the neck above the thyroid gland covered by skin without any signs of inflammation and/or trauma. The thyroid gland was apparently normal in size and consistence and no significant cervical adenopathy was found at physical examination. At entry serum chemistry tests, electrocardiagram and chest X-rays were normal. A neck ultrasonography identified a 4 cm cyst above a slightly enlarged thyroid gland without any significant alteration. A Sistrunk surgical procedure was then performed and a mass of 5 cm was removed including the entire duct from the gland to the level of the foramen cecum and the middle portion of the hyoid bone (Figure 1). A small 1 cm wide lymph node close to the cyst was also surgically removed. Post-operative follow-up was uneventful.

Gross examination of the surgical specimen showed a cystic mass of about 3 cm in greatest dimension with a smooth external surface. Microscopic examination showed the presence of papillary carcinoma with small areas of follicular carcinoma inside the thyroglossal duct cyst and metastatic disease in the adjacent lymph node. Further staging with neck sonogram showed an enlarged thyroid gland with a pattern suspicious for neoplastic disease subsequently confirmed by fine-needle aspiration biopsy. CT scan failed to identify distant metastatic disease. The patient underwent total nerve-sparing thyroidectomy with neck lymphoadenectomy. No significant post-surgical complications were recorded and the surgical wound healed regularly. Pathological examination showed a multinodular, moderately differentiated papillary and follicular carcinoma of the thyroid gland with focal invasion of the capsule and metastases in four neck lymph nodes. The main neoplastic nodule has a diameter of 1.8 cm. Post-operative staging according to the TNM classification was pT4b N1a M0. Surgical procedures were followed by iodine scan and radioactive iodine therapy with ^131^I ablation. Thyroid hormone replacement therapy was given regularly. In May 2003 evidence of iodine positive metastatic neck nodes was confirmed by fine-needle aspiration biopsy. Thyroglobulin levels were very high (355 ng/ml). The patient was rechallenged with ^131^I radioactive iodine therapy. To date the patient is still alive after 4 years.

## Discussion

Although some authors consider the thyroglossal duct as a natural way of spread of an occult thyroid cancer and also recommend total thyroidectomy with neck dissection, others believe that the malignancy arises directly in the thyroglossal duct cyst and consider surgical excision of the cyst as curative, at least in some cases [4,14,15]. The distinction between primary carcinoma arising from ectopic thyroid tissue and metastatic disease from an occult or manifest thyroid gland carcinoma is very important in therapeutic decision-making, but it is often difficult to achieve.

In this paper we have reported a case of TDC with concurrent thyroid gland carcinoma and locoregional lymph nodes metastases and we now discuss the surgical and medical treatments of this case.

To date a very limited number of cases of concomitant TDC and thyroid gland carcinoma have been reported in the medical literature [4]. Clinical presentation is that of a benign cyst with most patients showing an asymptomatic neck mass. Often the patient has noticed the mass many months or even years before presentation. A minority of patients may report pain, tenderness, hoarseness, dysphasia, sinus tract drainage and weight loss. In this paper we also report another case of a female with TDC and concomitant thyroid gland carcinoma with papillary and follicular pathology and metastatic disease to the locoregional lymph nodes. Full knowledge of the embryogenesis of the thyroid gland and the pathology of carcinomas arising in these structures is mandatory for the appropriate management of thyroglossal duct diseases [1,2,4]. Diagnosis relies on the pathologic analysis of the surgical specimen because the clinical presentation and evaluation of patients with TDC is indistinguishable from patients with benign disease [1]. This is not a hazard for patients owing to the fact that Sistrunk's surgical procedure is the optimal treatment for both TDC without thyroid gland neoplasm and simple thyroglossal duct cyst.

The pivotal problem concerning the treatment strategy is the management of the thyroid gland. While some authors consider Sistrunk's procedure adequate and curative in most cases, others suggests that a total thyroidectomy should be performed in case of TDC owing to the high incidence of associated papillary or mixed carcinomas in the thyroid gland [3,6]. Therefore significant attention must be paid to the pre-operative evaluation of a patient affected by a thyroglossal duct cyst, which should include a complete physical examination, accurate head and neck examination and palpation of the thyroid gland, thyroid function laboratory tests and also a thyroid scan. The presence of a firm mass in the thyroid gland or the cyst should raise the suspicion of malignancy although the incidence of malignancy is a thyroglossal duct cyst is estimated at around 1% of cases. Rapid enlargement may also induce suspicion, but infections in this area may also present a similar clinical picture. Although removal of the thyroglossal tract is a standard procedure, the actual extent of surgery depends on the degree of tumor involvement as detected through surgery or a thyroid scan. If the thyroid and lymph nodes are largely normal during Sistrunk's procedure, a post-operative thyroid scan is recommended [4]. If, however, a thyroid scan is abnormal or if either palpable thyroid nodules or a mass is identified during surgery, a thyroidectomy is recommended which may include also regional lymph node dissection.

Post-operative radioiodine ablative therapy should be given to patients with TDC and thyroid carcinoma. In the case of local recurrence or metastasic disease, further surgery, post-operative external-beam radiation or radioactive iodine therapy should be employed as needed. Data from medical literature suggest that squamous cell TDC appears to be more aggressive than other carcinomas and should be treated with post-operative bilateral external-beam radiotherapy [5,6]. As the thyroid tissue in a cyst is also sensitive to thyroid-stimulating hormone, pharmacological thyroid suppression with l-thyroxin is recommended for all patients with papillary TDC, independently of the presence of a normal thyroid scan or whether the patient underwent a thyroidectomy.

## Conclusion

This report adds another case of concurrent thyroglossal duct cyst carcinoma and thyroid carcinoma with metastases to metastatic lymph nodes to the medical literature. This case, according to previously reported data, strongly suggests the need for extensive thyroid evaluation in order to accurately plan surgical therapeutic strategy.

## Competing interests

The authors declare that they have no competing interests.

## Authors' contributions

CD performed the surgery, created the image of the specimen and wrote the surgical part of the case report. VG was in charge of patient's follow-up and treatment strategy and wrote the main body of the case report. MA participated in the diagnosis and therapy and wrote the endocrinology part of the case report. All authors read and approved the final manuscript.

## Consent

Written informed consent was obtained from the patient for publication of this case report and any accompanying images. A copy of the written consent is available for review by the Editor-in-Chief of this journal.
